# Physiological ecology meets climate change

**DOI:** 10.1002/ece3.1403

**Published:** 2015-02-05

**Authors:** Francisco Bozinovic, Hans-Otto Pörtner

**Affiliations:** 1 Departamento de Ecología, Center of Applied Ecology and Sustainability, Universidad Católica de ChileSantiago, Chile; 2 Alfred-Wegener-InstituteBremerhaven, Germany

**Keywords:** Adaptation, global warming, physiological diversity, plasticity, research programs, stress, tolerance, unifying concepts

## Abstract

In this article, we pointed out that understanding the physiology of differential climate change effects on organisms is one of the many urgent challenges faced in ecology and evolutionary biology. We explore how physiological ecology can contribute to a holistic view of climate change impacts on organisms and ecosystems and their evolutionary responses. We suggest that theoretical and experimental efforts not only need to improve our understanding of thermal limits to organisms, but also to consider multiple stressors both on land and in the oceans. As an example, we discuss recent efforts to understand the effects of various global change drivers on aquatic ectotherms in the field that led to the development of the concept of oxygen and capacity limited thermal tolerance (OCLTT) as a framework integrating various drivers and linking organisational levels from ecosystem to organism, tissue, cell, and molecules. We suggest seven core objectives of a comprehensive research program comprising the interplay among physiological, ecological, and evolutionary approaches for both aquatic and terrestrial organisms. While studies of individual aspects are already underway in many laboratories worldwide, integration of these findings into conceptual frameworks is needed not only within one organism group such as animals but also across organism domains such as Archaea, Bacteria, and Eukarya. Indeed, development of unifying concepts is relevant for interpreting existing and future findings in a coherent way and for projecting the future ecological and evolutionary effects of climate change on functional biodiversity. We also suggest that OCLTT may in the end and from an evolutionary point of view, be able to explain the limited thermal tolerance of metazoans when compared to other organisms.

## Introduction

Climate change occurs and becomes effective at global, regional, and local levels (IPCC 2014). Presently, temperature changes are evident, yet not similar, on all continents and in the oceans, causing shifts in species phenologies, physiological and behavioral traits, geographic ranges, productivity, and the disruption of diverse species interactions. Thereby, climate change causes pervasive effects on ecosystems (Parmesan 2006). Understanding the physiology of differential climate change effects on organisms is one of the many urgent challenges faced by contemporary science (Pörtner and Farrell 2008). For instance, responses of endotherms likely differ from those of ectotherms, those of mobile species differ from those of sedentary ones. Here, we explore further how physiological ecology can contribute to a holistic view of climate change impacts on organisms and ecosystems.

In several special volumes, ecological and evolutionary physiologists pointed out the increasing importance of studying the physiological basis of organism responses to climate change including their tolerance limits to environmental change (e.g., in, “Climate and Evolutionary Physiology,” Chown et al. 2010; “Effects of ocean acidification on marine ecosystems,” Browman et al. 2008; “Biological responses in an anthropogenically modified ocean,” Boyd and Hutchins 2012; “Survival in a Changing World,” Barnes et al. 2010). Physiological study requires identifying the differences in sublethal and lethal effects of climate change within and among species, as a precondition for the successful prediction of ecological effects which result from the success or failure of organisms, populations, and species to cope (e.g., Denny and Helmuth 2009; Somero 2011; Araujo et al. 2013). Indeed, individuals exposed to adverse climate change may reach a state that is beyond their capacity to maintain homeostasis and display performances such as growth, reproduction, and behaviors or to defend themselves against biotic and physicochemical stresses. Theoretical and experimental efforts not only need to improve our understanding of thermal limits to organisms, but also to consider effects of multiple drivers both on land (e.g., reduced water availability in warming terrestrial environments) and in the oceans (e.g., combined effect of ocean warming, acidification, and hypoxia). As a consequence of disturbances to organism functioning, fitness may be reduced, populations may lose genetic variation and collapse, and extinction becomes likely (Deutsch et al. 2008; Folguera et al. 2009). Understanding and explaining these phenomena must involve determining the combined and interactive effects of factors limiting the tolerances and distributions of species, by shaping the relevant physiological characteristics (Pörtner 2010; Bozinovic et al. 2011a). They must also involve knowledge of the scope of individual plasticity to shift such limits over time as well as the rate and limits of evolutionary adaptation to do so over generations (e.g., Reusch 2014).

## The Need for (a) Unifying Concept(s): OCLTT as an Example

Various conceptual and modeling approaches address relevant questions, for instance, one in thermal biology on how thermal reaction norms (performance curves) of organisms indicate limits to thermal tolerance (Angilletta 2009). However, these approaches usually do not identify the underlying physiological and biochemical mechanisms that are operative at various levels of biological organization. Recent efforts to understand climate sensitivity of marine ectotherms in the field led to the development of the concept of oxygen and capacity limited thermal tolerance (OCLTT) as a complex framework linking such levels from ecosystem to organism, tissue cell and molecular effects, and the effects of various drivers (Pörtner 2002, 2010, 2012). Essentially, the OCLTT concept aims to identify the mechanisms setting the shape and positioning of thermal reaction norms of species and their life stages on the temperature scale. The concept focuses on temperature as the key driving force in climate change impacts on biota, through temperature means, extremes, its changing variability as well as its interactions with other drivers. Here, we emphasize its ability to integrate thermal responses across levels of biological organization.

Including other drivers thus means assessing how and why such reaction norms respond to their effect. In the terrestrial realm, temperature interacts with water availability, atmospheric CO_2_, and nutrient levels. Ongoing distribution shifts of organisms are largely determined by the temperature trends in both the ocean (Poloczanska et al. 2013) and terrestrial climate; however, the capacity of organisms to move or restrain geographical barriers may restrain organisms to follow moving climate zones (Settele et al. 2014). Marine paleo-records suggest strong interference of temperature-induced changes with other drivers such as expanding hypoxia or ocean acidification (Pörtner et al. 2005). These effects combine with those of changing ocean currents and enhanced stratification regimes of warming oceans, which cause changes in nutrient availability and thus productivity of large ocean areas and gyres.

In addition to identifying the specific vulnerabilities and their variability within and between species, a key issue in understanding ecosystem shifts involves addressing how biotic interactions are modulated by climate, for example, through shifts along temperature-dependent performance curves of interacting organisms (Pörtner and Farrell 2008; Storch et al. 2014). As interactions involve organisms across all domains an overarching understanding of reaction norms is needed. It may build on the principle hypothesis that sublethal tolerance limits to temperature (influenced by other stressors) are set at the level of biological organization with the highest complexity. The associated limiting process(es) then involve(s) coordination of the largest number of body and cell compartments. In animals, the OCLTT concept proposes that the functional capacity of systems supplying and using oxygen sustains the aerobic performance capacity of the organism (Pörtner 2002; Pörtner and Farrell 2008) and becomes limiting at high temperature extremes. This hypothesis is supported by laboratory and field observations of, for example, declining growth and abundance in benthic eelpout, due to oxygen-dependent capacity limitations under extreme summer temperatures (Pörtner and Knust 2007). Declining exercise performance in migrating salmon sensitive to warming (Eliason et al. 2011) also reflects a key limiting role of cardiocirculatory capacity in covering demand.

Thermal ranges are principally wider in isolated molecules, organelles, or cells than in the intact organism. Molecular or organellar functions thus become thermally constrained earlier when integrated into the organism. The latter provides regulatory feedback on the use and expression of genome, transcriptome, and proteome such that these patterns follow the shape of the thermal reaction norm (Windisch et al. 2014), reflecting the mechanistic integration of molecular into whole organism functioning. Functional scope of the organism becomes constrained by limiting biotic interactions (e.g., limited food availability, competition, predatory pressure), which demand energy and reduce the energy excess supporting other life-sustaining performances and thus fitness. Such effects constrain the fundamental niche to the realized niche at ecosystem level and shrink the temperature-dependent geographical distribution of multicellular organisms such as animals and plants (Pörtner et al. 2010). These links and interdepencies across organizational levels, accessible through OCLTT, are only just emerging and need further investigation (Pörtner 2012). We are not aware of alternative concepts equally powerful in integrating molecular to whole organism and ecosystem functioning under climate change. We also suggest that recent attempts to prove or disprove the OCLTT framework have not been successful when using reductionist approaches outside ecological or evolutionary context. OCLTT may be common in animals using a convective oxygen supply system (thus possibly excluding adult insects). It has been proposed that more “classic” ways of interpreting the results of reductionist experiments (e.g., by Clark et al. 2013; Gräns et al. 2014; and Wang et al. 2014) do not capture relevant aspects and contrast the more integrative, ecosystem-oriented, and evolutionary ways of interpretation in accordance with OCLTT (cf. Farrell 2013; Pörtner and Giomi 2013; Pörtner 2014). For example, the term “capacity” is used differently by the OCLTT sceptics than by those who apply OCLTT. The former asks whether the circulatory system if pushed to its limits is able to provide sufficient oxygen to the organism until lethal temperatures (Gräns et al. 2014; Wang et al. 2014). The latter emphasize the interdependence of capacity and cost (e.g., of mitochondria, or cardiocirculation) and therefore variable maintenance costs and associated functional scope. Such cost is relatively low within the optimum but becomes overproportionally high toward the edge of the thermal tolerance range, then constraining functional scope (the percent increment with warming is highest in low capacity systems such as in polar or winter stenotherms, e.g., Pörtner, 2002, Wittmann et al. 2008). In this way, the progressive increase in thermal limitation is captured from the earliest onset of constraints to lethal temperatures. OCLTT might be viewed as an early evolutionary principle in animals that has been modified in various climate zones (e.g., polar areas, Pörtner et al. 2012) or during the evolution of air breathing (Giomi et al. 2015). This emphasizes the need for the parallel development of theoretical and experimental approaches, especially as experiments cannot resolve all facets of complex phenomena. Current theories of evolutionary biology illustrate these requirements (e.g., Angilletta 2009).

Lower complexity levels in most unicellular organisms thus support thermal limits higher than in animals. While the mechanisms causing limitations remain largely unexplored in this group, the complexity hypothesis leads to specific testable predictions: In archaea and bacteria, highest complexity levels may be found in molecular and/or membrane complexes with, for example, metabolic functions; in unicellular eukarya (heterotrophs), earliest functional limits may arise during coordination of mitochondria and cytosol. Eukaryotic phytoplankton with maximum thermal limits similarly low as in animals, possibly experience earliest limits during additional coordination of chloroplasts with mitochondria and the cytosol. This high cellular complexity may be an overarching constraint setting heat tolerance in plants (Pörtner 2002). Within each domain, however, individual species and life stages display differential thermal ranges reflecting specialization on temperature regimes, habitat characteristics, and mode of life. Heat limits can be modified by acclimation or adaptation at temperatures below domain-specific limits, but acclimation or evolutionary adaptation cannot overcome the specific limits of the domain as complexity cannot be changed. The OCLTT concept can serve as a role model for studying these principles across organism domains as needed for comprehensively understanding ecosystem level changes. We also suggest that OCLTT may in the end and from an evolutionary point of view, be a key concept able to explain the limited thermal tolerance of metazoans compared to other organisms.

From the point of view that the complexity principle underlying thermal specialization holds for all organisms in an ecosystem and defines differential, species-specific sensitivities, such specialization will also shape biotic interactions of coexisting species. Additional stressors such as ocean acidification may affect performances of interacting species differently due to divergent physiological ranges, optima, and sensitivities. This will change their fitness in predator prey or competitive interactions. Changes in performance thus produce changes in behaviors and phenologies, and thereby the balance and synchronization of trophic levels and species community structure (Pörtner and Farrell 2008; Bozinovic et al. 2013b).

Further joint studies by physiologists and ecologists should thus look at species interactions across domains and climate zones, as well as in various, aquatic and terrestrial ecosystems. Overarching mechanistic frameworks, such as OCLTT for animals, need to be developed for all organisms and complemented, for example, under scenarios of warming, hypoxia, and acidification in the ocean, or under drought and changing temperature means, extremes, and variability on land.

## Toward a Unifying Experimental Approach

An important shortcoming of previous approaches has been that experimental laboratory and field studies mostly tested one variable at a time. Additive or synergistic effects of various drivers, however, have occurred under climate change in the past and also characterize ongoing climate change (Pörtner et al. 2005), for example, in the ocean (e.g., Boyd and Hutchins 2012; Pörtner 2012; Pörtner et al. 2014). OCLTT illustrates a new way how to integrate different drivers and traits (Pörtner 2010) or how to parameterize fundamental traits for the modeling of functional limits, diseases, species abundances, biogeographical range. Increased variability and frequency of extreme events under climate change require consideration (Rahmstorf and Coumou 2011). Studies in thermal biology have often focused on the impact of shifting mean values on organisms but temperature variability may also act as a selective force (Pörtner and Knust 2007; Bozinovic et al. 2013a; Clavijo-Baquet et al. 2014). The development of a widely accepted theoretical basis should relate physiology to population processes and address the role of temperature means and variability at higher ecological levels and their potentially nonadditive interaction shaping performance.

We therefore summarize the following core objectives of a comprehensive research program comprising ecophysiological approaches, acknowledging that studies of individual aspects are already underway in many laboratories worldwide. Most importantly, once results become available, they would need to be interpreted in the context of conceptual frameworks such as OCLTT in animals and others that still need to be developed across organism domains:

1. To determine the effects of isolated and coupled climate variables at physiological levels of different taxonomic groups; and to determine the bases of sublethal and lethal stress and capacities to respond to stresses induced by climate.
2. To explore the physical, chemical, and biotic factors that affect and limit the distribution of species. At distribution limits, these factors may well indicate some of the likely consequences of climate change in the future. Such analyses may be most important in species that face extreme conditions today.
3. To study the relationship between tolerances and climate in scenarios of climate change; as well as to analyze how trade-offs in genetic adaptation may set limits to distribution and abundance.
4. To assess how phenotypic traits are affected by high levels of environmental variability encountered over large geographic distances, but also over long time scales.
5. To identify how physical, chemical, and biotic factors linked to climate change induce noxious phenotypic states in some species but not in others, and shape the distribution and coexistence of species.
6. To test how trophic interactions (e.g., plant–herbivore) are affected by changes in temperature and how it may impact community structure and function. In this case, an herbivore may alter how species respond to raised temperatures. This dependency in the response of both herbivore and plant to temperature suggests that the ecological impacts of future climate change on trophic interactions may be an import avenue of research.
7. To determine the limits and pathways of genetic adaptation, phenotypic plasticity, and life-history strategies to climate change and to analyze how different life-history stages are affected by climate variables.
8. To improve the capacity of modeling tools used to predict effects of climate on species distributions and abundances, by incorporating physiological and molecular information.

Ecologists would need to consider this framework when developing different models of how functional biodiversity can be impacted by climate change. Many of them concur that these models lack or oversimplify the physiological basis of climate change responses, which may lead to very large under- or overestimations of risks and vulnerabilities for individuals, species, and ecosystems (Huey et al. 2009). Thus, research programs should incorporate physiological approaches, also when exploring conservation, restoration, and management.

The IPCC (2014) concluded that during the course of this century, the ability of many organisms and ecosystems to adapt naturally is likely to be surpassed by an unprecedented rate of climate change, an unprecedented combination of changes in climate, associated extreme events, and anthropogenic influences such as pollution, eutrophication, overexploitation, land use, and other disturbances. As a consequence, nearly 20–30% of biodiversity are at increasing risk of extinction as global mean temperatures increase by 2 to 3°C above preindustrial levels. Our proposal is that a solid and mechanistic foundation is required for developing projections of probable outcomes under climate change (Estay et al. 2014; Pörtner et al. 2014). Development of unifying concepts such as OCLTT is relevant for interpreting existing and future findings in a coherent way and for projecting the future ecological and evolutionary effects of climate change on whole-organism and ecosystem functioning (Bozinovic et al. 2011a,b, 2013a,b; Folguera et al. 2011; Pörtner et al. 2014).

## References

[b1] Angilletta MJ (2009). Thermal adaptation: a theoretical and empirical synthesis.

[b2] Araujo MB, Ferri-Yañez F, Bozinovic F, Chown SL, Marquet PA, Valladares F (2013). Heat freezes niche evolution. Ecol. Lett.

[b3] Barnes B, Gordon M, Sato eds K (2010). Surviving in a changing world. J. Exp. Biol.

[b4] Boyd PW, Hutchins DA (2012). Biological responses in an anthropogenically modified ocean. Mar. Ecol. Prog. Ser.

[b5] Bozinovic F, Bastías DA, Boher F, Clavijo-Baquet S, Estay SA, Angilletta MJ (2011a). The mean and variance of environmental temperature interact to determine physiological tolerance and fitness. Physiol. Biochem. Zool.

[b6] Bozinovic F, Calosi P, Spicer JI (2011b). Physiological correlates of geographic range in animals. Annu. Rev. Ecol. Evol. Syst.

[b7] Bozinovic F, Catalán TP, Kalergis AM (2013a). Immunological vulnerability and adjustments to environmental thermal variability. Rev. Chil. Hist. Nat.

[b8] Bozinovic F, Catalán TP, Estay SA, Sabat P (2013b). Acclimation to daily thermal variability drives the metabolic performance curve. Evol. Ecol. Res.

[b9] Browman HI, Vézina AF, Hoegh-Guldberg O (2008). Effects of ocean acidification on marine ecosystems. Mar. Ecol. Prog. Ser.

[b10] Chown SL, Hoffmann AA, Kristensen TN, Angilletta MJ, Stenseth NC, Pertoldi C (2010). Adapting to climate change: a perspective from evolutionary physiology. Clim. Res.

[b11] Clark TD, Sandblom E, Jutfelt F (2013). Aerobic scope measurements of fishes in an era of climate change: respirometry, relevance and recommendations. J. Exp. Biol.

[b12] Clavijo-Baquet S, Boher F, Ziegler L, Martel SI, Estay SA, Bozinovic F (2014). Differential responses to thermal variation between fitness metrics. Sci. Rep.

[b13] Denny M, Helmuth B (2009). Confronting the physiological bottleneck: a challenge from ecomechanics. Int. Comp. Biol.

[b14] Deutsch CA, Tewksbury JJ, Huey RB, Sheldon KS, Ghalambor CK (2008). Impacts of climate warming on terrestrial ectotherms across latitude. Proc. Natl Acad. Sci. USA.

[b15] Eliason EJ, Clark TD, Hague MJ, Hanson LM, Gallagher ZS, Jeffries KM (2011). Differences in thermal tolerance among sockeye salmon populations. Science.

[b16] Estay SA, Lima M, Bozinovic F (2014). The role of temperature variability on insect performance and population dynamics in a warming world. Oikos.

[b17] Farrell AP (2013). Aerobic scope and its optimum temperature: clarifying their usefulness and limitations. J. Exp. Biol.

[b18] Folguera G, Bastías DA, Bozinovic F (2009). Impact of experimental thermal amplitude on ectotherm performance: adaptation to climate change variability?. Comp. Biochem. Physiol. A.

[b19] Folguera G, Bastías DA, Caers J, Rojas JM, Piulachs MD, Bellés X (2011). An experimental test of the role of environmental temperature variability on ectotherm molecular, physiological and life-history traits: implications for global warming. Comp. Biochem. Physiol.

[b20] Giomi F, Fusi M, Barausse A, Mostert B, Pörtner HO, Cannicci S (2015). Improved heat tolerance in air drives the recurrent evolution of air-breathing. Proc. R. Soc. B.

[b21] Gräns A, Jutfelt F, Sandblom E, Jönsson E, Wiklander K, Seth H (2014). Aerobic scope fails to explain the detrimental effects on growth resulting from warming and elevated CO_2_ in Atlantic halibut. J. Exp. Biol.

[b23] Huey RB, Deutsch CA, Tewksbury JJ, Vitt LJ, Hertz PE (2009). Why tropical forest lizards are vulnerable to climate warming. Proc. R. Soc. Lond. B.

[b24] Field CB, IPCC (2014). Impacts, adaptation, and vulnerability. Contribution of working group II to the Fifth assessment report of the Intergovernmental panel on climate change.

[b25] Parmesan C (2006). Ecological and evolutionary responses to recent climate change. Annu. Rev. Ecol. Evol. Syst.

[b26] Poloczanska ES, Brown CJ, Sydeman WJ, Kiessling W, Schoeman DS, Moore PJ (2013). Global imprint of climate change on marine life. Nat. Clim. Chang.

[b27] Pörtner HO (2002). Physiological basis of temperature dependent biogeography: trade-offs in muscle design and performance in polar ectotherms. J. Exp. Biol.

[b28] Pörtner HO (2010). Oxygen and capacity limitation of thermal tolerance: a matrix for integrating climate related stressors in marine ecosystems. J. Exp. Biol.

[b29] Pörtner HO (2012). Integrating climate-related stressor effects on marine organisms: unifying principles linking molecule to ecosystem-level changes. Mar. Ecol. Prog. Ser.

[b30] Pörtner HO (2014). How to and how not to investigate the oxygen and capacity limitation of thermal tolerance (OCLTT) and aerobic scope. J. Exp. Biol.

[b31] Pörtner HO, Farrell AP (2008). Physiology and climate change. Science.

[b32] Pörtner HO, Giomi F (2013). Nothing in experimental biology makes sense except in the light of ecology and evolution - correspondence on. J. Exp. Biol.

[b33] Pörtner HO, Knust R (2007). Climate change affects marine fishes through the oxygen limitation of thermal tolerance. Science.

[b34] Pörtner HO, Langenbuch M, Michaelidis B (2005). Synergistic effects of increased CO_2_, temperature and hypoxia on marine animals. J. Geo. Res.

[b35] Pörtner HO, Schulte PM, Wood CM, Schiemer F (2010). Niche dimensions and limits in fishes: an integrative view. Illustrating the role of physiology in understanding ecological realities. Physiol. Biochem. Zool.

[b36] Pörtner HO, Karl DM, Boyd PW, Cheung WL, Lluch-Cota SE, Nojiri Y, Field CB (2014). Ocean systems. Climate change 2014: impacts, adaptation, and vulnerability. Part A: global and sectoral aspects. Contribution of working group II to the Fifth assessment report of the Intergovernmental panel on climate change.

[b37] Rahmstorf S, Coumou D (2011). Increase of extreme events in a warming world. Proc. Natl Acad. Sci. USA.

[b38] Reusch TBH (2014). Climate change in the oceans: evolutionary vs. phenotypically plastic responses in marine animals and plants. Evol. Appl.

[b40] Settele J, Scholes R, Betts R, Bunn S, Leadley P, Nepstad D, Field CB (2014). Terrestrial and inland water systems. Climate change 2014: impacts, adaptation, and vulnerability. Part A: global and sectoral aspects. Contribution of working group II to the Fifth assessment report of the Intergovernmental panel on climate change.

[b41] Somero GN (2011). Comparative physiology: a “crystal ball” for predicting consequences of global change. Am. J. Physiol.

[b42] Storch D, Menzel L, Frickenhaus S, Pörtner HO (2014). Climate sensitivity across the domains of life: limits to evolutionary adaptation shape species interactions. Glob. Chang. Biol.

[b43] Wang T, Lefevre S, Iversen NK, Findorf I, Buchanan R, McKenzie DJ (2014). Anaemia only causes a small reduction in the upper critical temperature of sea bass: is oxygen delivery the limiting factor for tolerance of acute warming in fishes?. J. Exp. Biol.

[b44] Windisch HS, Frickenhaus U, John R, Knust R, Pörtner HO, Lucassen M (2014). Stress response or beneficial temperature acclimation: transcriptomic signatures in Antarctic fish (*Pachycara brachycephalum*. Mol. Ecol.

[b45] Wittmann A, Schröer M, Bock C, Steeger H-U, Paul R, Pörtner HO (2008). Seasonal patterns of thermal tolerance and performance capacity in lugworm (*Arenicola marina*) populations in a latitudinal cline. Clim. Res.

